# Zoster vaccination inequalities: A population based cohort study using linked data from the UK Clinical Practice Research Datalink

**DOI:** 10.1371/journal.pone.0207183

**Published:** 2018-11-15

**Authors:** Anu Jain, Jemma L. Walker, Rohini Mathur, Harriet J. Forbes, Sinéad M. Langan, Liam Smeeth, Albert J. van Hoek, Sara L. Thomas

**Affiliations:** 1 Faculty of Epidemiology and Population Health, London School of Hygiene and Tropical Medicine, London, United Kingdom; 2 Statistics, Modelling and Economics Department, Public Health England, London United Kingdom; Liverpool John Moores University, UNITED KINGDOM

## Abstract

**Objective:**

To quantify inequalities in zoster vaccine uptake by determining its association with socio-demographic factors: age, gender, ethnicity, immigration status, deprivation (at Lower-layer Super Output Area-level), care home residence and living arrangements.

**Method:**

This population-based cohort study utilised anonymised primary care electronic health records from England (Clinical Practice Research Datalink) linked to deprivation and hospitalisation data. Data from 35,333 individuals from 277 general practices in England and eligible for zoster vaccination during the two-year period (2013–2015) after vaccine introduction were analysed. Logistic regression was used to obtain adjusted odds ratios (aOR) for the association of socio-demographic factors with zoster vaccine uptake for adults aged 70 years (main target group) and adults aged 79 years (catch-up group).

**Results:**

Amongst those eligible for vaccination, 52.4% (n = 18,499) received the vaccine. Socio-demographic factors independently associated with lower zoster vaccine uptake in multivariable analyses were: being older (catch-up group: aged 79 years) aOR = 0.89 (95% confidence interval (CI):0.85–0.93), care home residence (aOR = 0.64 (95%CI: 0.57–0.73)) and living alone (aOR = 0.85 (95%CI: 0.81–0.90)). Uptake decreased with increasing levels of deprivation (p-value for trend<0.0001; aOR most deprived versus least deprived areas = 0.69 (95%CI: 0.64–0.75)). Uptake was also lower amongst those of non-White ethnicities (for example, Black versus White ethnicity: aOR = 0.61 (95%CI: 0.49–0.75)) but was not lower among immigrants after adjusting for ethnicity. Lower uptake was also seen amongst females compared to men in the catch-up group.

**Conclusions:**

Inequalities in zoster vaccine uptake exist in England; with lower uptake among those of non-White ethnicities, and among those living alone, in a care home and in more deprived areas. Tailored interventions to increase uptake in these social groups should assist in realising the aim of mitigating vaccination inequalities. As care home residents are also at higher risk of zoster, improving the uptake of zoster vaccination in this group will also mitigate inequalities in zoster burden.

## Introduction

Zoster is caused by reactivation of latent varicella-zoster virus infection and mainly affects older individuals. It is characterised by a painful dermatomal rash which may be followed by persisting pain called post-herpetic neuralgia (PHN).[1] Amongst individuals aged ≥70 years in England and Wales, an estimated ~53,000 cases of zoster occur annually of which ~27% develop post-herpetic neuralgia.[2] To reduce zoster disease burden, the UK introduced a national zoster vaccination programme (using a live vaccine: Zostavax manufactured by Merck and Co. Inc., USA) in 2013, targeting individuals aged 70 years, with a catch-up programme targeting older age groups.[3–5] The programme comprises vaccine administration to individuals aged 70 years on 1 September of the corresponding year (the routine cohort). For the catch-up programme, the vaccine was gradually rolled out in 2013/14 to individuals aged 79 years on 1 September 2013, and in 2014/15 to those aged 78 and 79 years on 1 September 2014.[6, 7] Additionally, eligible individuals who missed the vaccine in previous years were given the opportunity to get vaccinated in subsequent years. At introduction, uptake of the programme was around ~62% in the routine cohort but has decreased to ~55% in 2015–2016.[8] The reasons cited for this decline include difficulties experienced by general practice personnel who were busy with seasonal influenza vaccination, challenges in identifying individuals eligible for vaccination, insufficient follow-up of unvaccinated individuals and a potential decline in vaccine knowledge amongst the eligible cohort.[6–8]

Monitoring and reducing inequalities in healthcare services or interventions is a statutory requirement in the UK.[9] Inequalities in vaccine uptake, resulting in higher disease burden in specific population groups, are well described.[10–13] Our 2017 systematic review and meta-analysis investigated vaccine uptake amongst individuals aged ≥65 years in Europe and reported lower seasonal influenza vaccine uptake amongst individuals living alone, those residing in more deprived areas and amongst immigrants.[12] Currently the national zoster post-vaccination surveillance in England comprises collection of aggregated general practice level data with information only on gender and limited ethnicity data.[8] The national zoster vaccine uptake for England was found generally to be higher amongst males, particularly in the catch-up cohort.[8] The aggregated national zoster uptake data were also utilised in a 2017 study, which reported lower zoster vaccine uptake in deprived areas and amongst most non-White ethnic groups.[14] However in this study, deprivation was assessed as an ecological factor and individuals were assigned ethnicity and vaccination status derived from the proportions reported only at an aggregated general practice level.[14]

Ascertainment of the socio-demographic determinants of zoster vaccine uptake can provide important information to public health professionals to address vaccination-related inequalities and reduce zoster disease burden. We have recently shown that routinely collected clinical and administrative information in the form of anonymised linked electronic health records are a useful resource to examine some of these socio-demographic factors, and these data can be used to supplement the routine surveillance data.[15]

The primary objective of this study was therefore to identify the socio-demographic determinants, of zoster vaccine uptake in England, using one of the world’s largest databases of general practice electronic health records: the Clinical Practice Research Datalink (CPRD),[16] with an overarching aim of mitigating vaccination inequalities amongst older individuals. The nine socio-demographic factors of interest included: age, gender, ethnicity, immigration status, deprivation (patient- and practice-small area-level), marital status, cohabitation, living alone and care home residence. As a secondary objective, we also ascertained inadvertent zoster vaccination of individuals whilst they were immunosuppressed, to quantify possible violations of the inclusion criteria.

## Methods

### Data source

The CPRD provides quality-assured anonymised primary care patients’ clinical, administrative and lifestyle data representative of the UK population and covering approximately 7% of general practices from England.[16, 17] Additionally, ~75% of CPRD general practices based in England can be linked at an individual level to hospitalisation (Hospital Episode Statistics, HES) data,[18] which provides information on all admissions to NHS hospitals, and at the Lower-layer Super Output Area (LSOA) level [19] to deprivation data (Index of Multiple deprivation (IMD) score).[19, 20] The English IMD score is a composite measure of relative deprivation for small geographic areas (LSOA), which cover an average population of 1500 individuals or 650 households.[19] This score is derived from using seven domains of deprivation: education, employment, income, health and disability, crime, housing and living environment with no definitive cut-off points for defining deprivation.[19] The validity of various diagnoses recorded in CPRD was reported as high in a systematic review spanning a 21-year study period.[21]

### Study population

This 2-year cohort study from England spanned the period from 01/09/2013 to 31/08/2015, the first two years after the zoster vaccine was introduced. To maintain patient anonymity, CPRD data provide only year of birth for adult patients. This posed a problem in how to identify individuals who were eligible for zoster vaccination, which is determined by their age on a specific date. The common convention of using the mid-year (1^st^ July) to assign study participants’ day and month of birth would wrongly classify some individuals as eligible for zoster vaccination. Importantly, the resulting unvaccinated group would comprise a mixture of individuals with possibly differing social factors: a) those eligible for vaccination who chose not to be vaccinated and b) those ineligible on the grounds of age, thus potentially resulting in biased effect estimates. To address this, we selected all individuals born in 1943 (or 1934 for catch-up cohort), who would have been eligible for vaccination at some point during the 2-year follow-up period as follows: those born in January-August 1943 would be eligible for the vaccine in 2013/14 or in 2014/15 if born September-December 1943; and determined vaccine uptake for the 2-year study period. The study population therefore comprised individuals born in 1943 (the routine cohort) and in 1934 (the catch-up cohort), who were alive and registered on 01/09/2013 (the start of the national programme) with a CPRD general practice that had agreed to linkage to HES and IMD data and that met CPRD’s quality assurance criteria.[20] Start of follow up was on 01/09/2013 and a minimum of five months of follow-up was required from then (i.e. from September until the end of January, coinciding with the main part of the seasonal influenza vaccination season),[22] to ensure that individuals had sufficient opportunity to receive zoster vaccination. Individuals who had any immunosuppressive conditions or therapies at the start of follow up, that were contraindications to receiving the live zoster vaccine,[4] were excluded from analyses of the socio-demographic determinants of vaccine uptake but included in descriptive analyses of inadvertent zoster vaccinations amongst immunosuppressed individuals. All individuals with zoster vaccine codes prior to the start of national programme and start of the study (01/09/2013) were also excluded.[23, 24] End of follow-up was defined as the earliest of: (a) the end of the study (31/08/2015), (b) individuals’ transfer out date from the practice, (c) individuals’ date of death, or (d) the date data were last collected from the practice.[20]

### Defining the outcome

Zoster vaccination was determined in five different data files in CPRD: using product codes in patients’ therapy files, immunisation codes in their immunisation files and Read codes (S1 Table) in their clinical, referral and test files.[20] Additional immunisation and Read codes provided further information on whether the vaccine was advised, refused or administered. When vaccination data appeared in more than one file, we used an algorithm to assign vaccination date for each individual and handle conflicting information; details are provided in the S1 Text and S1 Fig.

### Exposure variables

The socio-demographic factors of interest were identified based on our previously developed methodology of using CPRD linked to HES and IMD data.[15] The factors of interest, in addition to age and gender, included ethnicity, immigration status, care home residence, marital status, cohabitation (defined as two individuals living as a couple) and living alone; code lists are provided in S2 Table. The latter three social factors provided complementary information about an individual’s living arrangements. Religion was not examined as our previous work has shown it to be poorly recorded in CPRD data (<3% of older individuals).[15] For binary socio-demographic variables, individuals without relevant codes were considered not to have that characteristic. Ethnicity (five categories: White, South Asian, Black, Others and Mixed) and immigration status (binary) were defined as factors that did not vary with time. Time-varying factors included marital status (six categories: single, widow, married, divorced, separated, partner uncategorised/other), cohabitation, living alone and care home residence (binary variables). All the time-varying factors were determined at the start of follow up, with any changes by the end of the 2-year follow-up period quantified and described. Actual IMD scores are not made available to researchers by CPRD, to avoid identification of patients’ area of residence, but all LSOA-level IMD scores in England are ranked (with scores in 2015 ranging from 0.47 to 92.6) and then divided into quintiles for research use. Deprivation data (IMD quintile at LSOA level: 2015, quintile 1 representing least deprived and quintile 5 the most deprived quintile of deprivation) for both patient- and practice-LSOA-level l were available. Practice- LSOA-level IMD quintiles were used if patient-level data were unavailable.

### Other variables

At the initiation of the zoster vaccination programme, general practitioners (GPs) were encouraged to co-administer zoster with seasonal influenza vaccine (SIV).[25] We therefore investigated the concurrent administration of zoster vaccine with SIV. This was achieved by identifying specific product codes, immunisation type codes and Read codes in CPRD (S3 Table) during the SIV campaign season (September-March)[26] of 2013/14 and 2014/15. Individuals who received SIV or/and zoster vaccine were quantified.

We also identified, throughout the study period, individuals who had immunosuppressive conditions or treatments that were contraindications to receipt of zoster vaccine. This was done to identify those who were eligible to receive the live zoster vaccine for the main analysis, and to describe the extent of inadvertent administration of zoster vaccine to those with contraindications. The immunosuppressive conditions and drugs included were those listed in the UK Green Book; code lists (S2 Table) and algorithms used to identify these are described in the S4 Table.[4]

A past history of zoster was also ascertained using zoster or PHN codes from both CPRD and HES (S5 Table) occurring before the start of follow-up (01/09/2013), or a first zoster code of PHN occurring during follow-up.

### Analyses

A complete case analysis using multivariable logistic regression was used to determine the association of socio-demographic factors with zoster vaccine uptake, using adjusted odds ratios (aOR) with 95% confidence intervals (CI). Logistic regression models were chosen to address the problem of potential misclassification of individuals for vaccine eligibility based on their date of birth and therefore the lack of information on person-time at risk for vaccination. The exposure and outcome characteristics of individuals excluded from complete case primary analysis because of missing data were described.

Gender and being a part of the routine or catch-up cohort (born in 1943 and 1934, respectively) were considered to be *a priori* confounders of the socio-demographic factors of interest, as well as potential determinants of zoster vaccine uptake. An existing conceptual framework[27] was adapted to postulate the hierarchical inter-relationships between distal and proximate social factors with the outcome (S6 Table).[28] Using this framework, socio-demographic factors were added sequentially, as long as data sparsity or multicollinearity were not encountered. The three multivariable models were as follows: Model 1 adjusted for a priori confounders (gender and year of birth) immigration status and ethnicity; Model 2 additionally adjusted for deprivation, and Model 3 additionally adjusted for the remaining socio-demographic factors. Standard errors of the coefficients were compared in successive analyses to assess multicollinearity between socio-demographic factors. Likelihood ratio tests were conducted for hypothesis testing unless otherwise indicated. Linear trends, if appropriate, were also examined for ordered categorical variables such as patient- and practice-LSOA-level IMD. The catch-up programme is different from the routine programme, and differences between the two age groups (routine and catch-up) in the effect of one sociodemographic factor (gender) on vaccine uptake have been reported by the national surveillance data.[6, 7] We therefore investigated whether the effects of the social factors varied between the two age cohorts by adding interaction terms to the models.

In sensitivity analyses, we repeated multivariable analyses: 1) restricted to individuals who were followed up for the entire study period of two years; 2) adding the social factors that had been excluded due to multicollinearity issues; 3) replacing the status of the time-varying factors to that ascertained at the end of follow-up instead of at the start of follow-up, and 4) including past history of zoster in the final multivariable model as a potential mediator of some of the socio-demographic factors.

Additionally, inadvertent zoster vaccination amongst individuals with immunosuppressive conditions or therapies was also described.

Data were analysed using Stata 14 (StataCorp, College Station, TX, USA).

### Ethics

Approval was sought and obtained from the Observational/Interventions Research Ethics Committee of the London School of Hygiene and Tropical Medicine (Reference: 11910) and from the Independent Scientific Advisory Committee (ISAC) of the Medicines and Healthcare products Regulatory Agency (reference:16_168). The ISAC protocol was made available to the reviewers of this paper.

## Results

**Fig 1:**
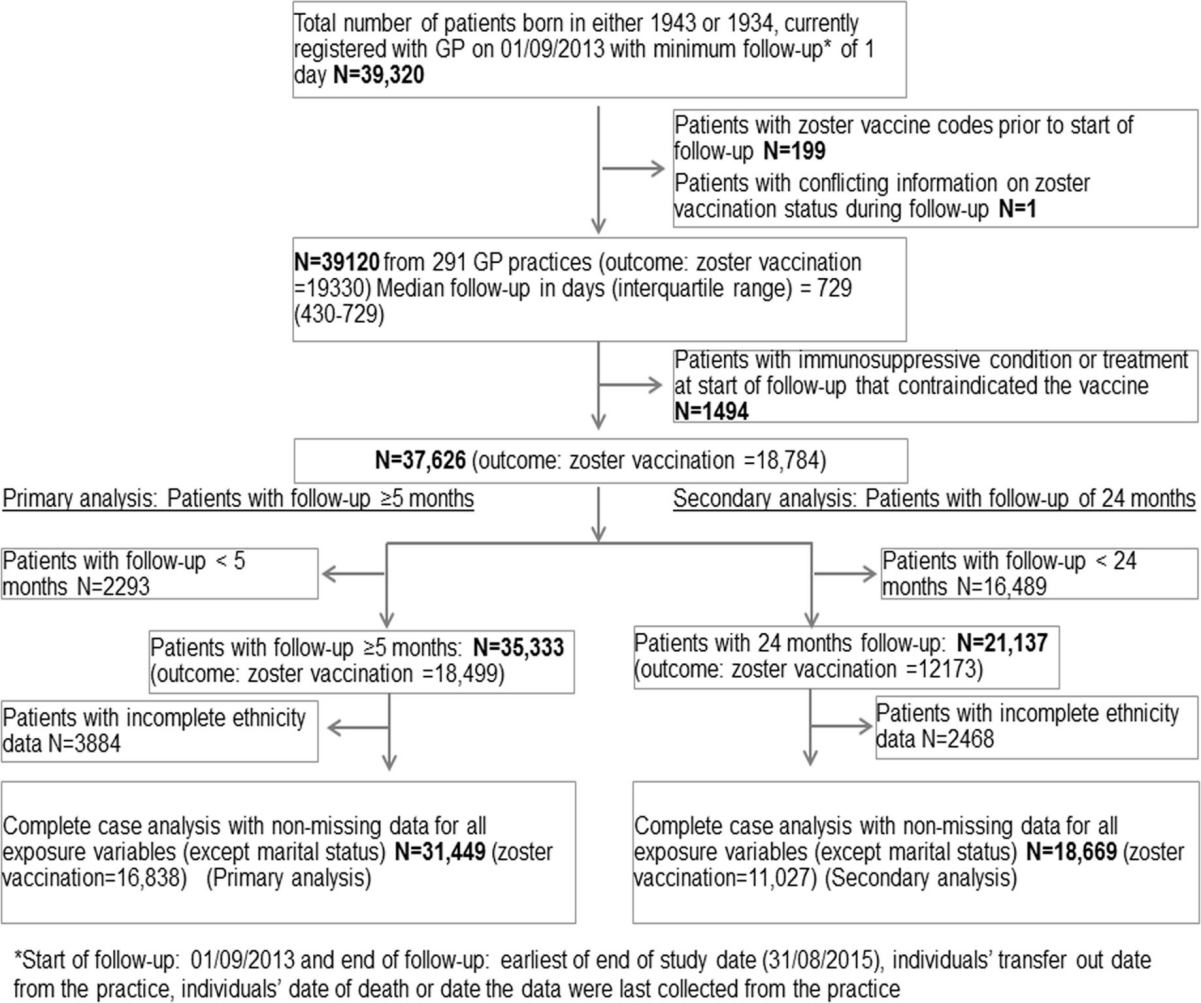
Zoster vaccine uptake study flow chart.

A total of 39,120 individuals born in 1943 or 1934 and with no evidence of prior zoster vaccination were registered with a CPRD practice, which had consented to linkages, on 01/09/2013 (Fig 1). The percentage of individuals who had relevant codes for binary socio-demographic variables (as described in the Methods) was 1.8% for immigration status, 9.2% for care home residence, 63.6% for cohabitation and 72.4% for living alone variable. After excluding those who had contraindications to zoster vaccine at the start of follow-up or less than 5 months follow-up, 35,333 individuals from 277 practices were considered for the primary analysis (Fig 1).

### Primary analysis

A slight majority of the participants in the primary analysis (Table 1) were female. A higher proportion was born in the year 1943 (the main target group), were from a White ethnic background, and were cohabiting (living as a couple) and/or not living alone. Data for marital status were missing for 37.1% participants (Table 1) and practice-LSOA-level IMD was used to replace the missing patient-LSOA-level IMD for 0.07% (n = 26) participants. A past history of zoster was present for 11.4% of participants (Table 1).

**Table 1:** Baseline characteristics of the study population: Comparison of individuals with minimum follow-up of 5 months and 24 months.

Variables		Primary analysis^a^ N = 35333 from 277 practices, vaccine uptake = 18499 (52.4%) N (%) registered with their practice for ≥6months on 01/09/2013 = 34828 (98.6%) Median duration (months) of registration on 01/09/2013 (IQR): 255.8 (IQR 137–402.4)		Sensitivity analysis^b^ N = 21137 from 178 practices, vaccine uptake = 12173 (57.6%) N (%) registered with their practice for ≥6months on 01/09/2013 = 20848 (98.6%) Median duration (months) of registration on 01/09/2013 (IQR): 263.8 (IQR 145–396.1)	
		Total (column %)	Received zoster vaccine (row %)	Total (column %)	Received zoster vaccine (row %)
Gender	Male	16633 (47.1%)	8859 (53.3%)	9829 (46.5%)	5763 (58.6%)
	Female	18700 (52.9%)	9640 (51.6%)	11308 (53.5%)	6410 (56.7%)
Year of birth	1943	21458 (60.7%)	11452 (53.4%)	13011 (61.6%)	7580 (58.3%)
	1934	13875 (39.3%)	7047 (50.8%)	8126 (38.4%)	4593 (56.5%)
Immigration status	Not immigrant	34821 (98.6%)	18270 (52.5%)	20891 (98.8%)	12052 (57.7%)
	Immigrant	512 (1.4%)	229 (44.7%)	246 (1.2%)	121 (49.2%)
Ethnicity	White	30052 (85.1%)	16244 (54.1%)	18044 (85.4%)	10709 (59.3%)
	South Asian	669 (1.9%)	304 (45.4%)	269 (1.3%)	136 (50.6%)
	Black	380 (1.1%)	147 (38.7%)	172 (0.8%)	89 (51.7%)
	Other	262 (0.7%)	107 (40.8%)	138 (0.7%)	68 (49.3%)
	Mixed	86 (0.2%)	36 (41.9%)	46 (0.2%)	25 (54.3%)
	Missing	3884 (11%)	1661 (42.8%)	2468 (11.7%)	1146 (46.4%)
Patient-LSOA-level IMD^~#^	Least deprived	9313 (26.4%)	5230 (56.2%)	5521 (26.1%)	3429 (62.1%)
	2	8692 (24.6%)	4670 (53.7%)	5096 (24.1%)	2959 (58.1%)
	3	7520 (21.3%)	3884 (51.6%)	4644 (22%)	2645 (57%)
	4	5828 (16.5%)	2890 (49.6%)	3595 (17%)	1950 (54.2%)
	Most deprived	3980 (11.3%)	1825 (45.9%)	2281 (10.8%)	1190 (52.2%)
Practice-LSOA-level IMD	Least deprived	6184 (17.5%)	3479 (56.3%)	3190 (15.1%)	1922 (60.3%)
	2	7979 (22.6%)	3952 (49.5%)	4994 (23.6%)	2711 (54.3%)
	3	7407 (21%)	3849 (52%)	4157 (19.7%)	2464 (59.3%)
	4	6455 (18.3%)	3488 (54%)	4040 (19.1%)	2321 (57.5%)
	Most deprived	7308 (20.7%)	3731 (51.1%)	4756 (22.5%)	2755 (57.9%)
Care home*	No	34133 (96.6%)	17976 (52.7%)	20509 (97%)	11851 (57.8%)
	Yes	1200 (3.4%)	523 (43.6%)	628 (3%)	322 (51.3%)
Living alone*	No	25525 (72.2%)	13738 (53.8%)	15419 (72.9%)	9122 (59.2%)
	Yes	9808 (27.8%)	4761 (48.5%)	5718 (27.1%)	3051 (53.4%)
Cohabiting*	No	15352 (43.4%)	7316 (47.7%)	8899 (42.1%)	4691 (52.7%)
	Yes	19981 (56.6%)	11183 (56%)	12238 (57.9%)	7482 (61.1%)
Marital status*	Single	497 (1.4%)	232 (46.7%)	295 (1.4%)	148 (50.2%)
	Married/Civil	6495 (18.4%)	3502 (53.9%)	3959 (18.7%)	2372 (59.9%)
	Widow/er	1537 (4.4%)	800 (52%)	872 (4.1%)	508 (58.3%)
	Divorced	516 (1.5%)	246 (47.7%)	304 (1.4%)	163 (53.6%)
	Separated	143 (0.4%)	64 (44.8%)	93 (0.4%)	50 (53.8%)
	Partner other/ uncategorised	13091 (37.1%)	7465 (57%)	8028 (38%)	4969 (61.9%)
	Missing	13054 (36.9%)	6190 (47.4%)	7586 (35.9%)	3963 (52.2%)
History of zoster*	No	31319 (88.6%)	16286 (52%)	18732 (88.6%)	10721 (57.2%)
	Yes	4014 (11.4%)	2213 (55.1%)	2405 (11.4%)	1452 (60.4%)

^a^Those with immunosuppressing condition at start of follow-up excluded with minimum follow-up > = 5 months LSOA Lower-layer Super Output Area IMD Index of Multiple Deprivation: a composite measure of relative deprivation for LSOA-level (details in the text) IQR interquartile range ~ 26 and ^**#**^2 patients with missing patient-LSOA-level IMD were replaced with practice-LSOA-level IMD for primary and secondary analyses respectively

^b^Those with immunosuppressing condition at start of follow-up excluded with minimum follow-up > = 24 months

*at start of follow-up

**Fig 2:**
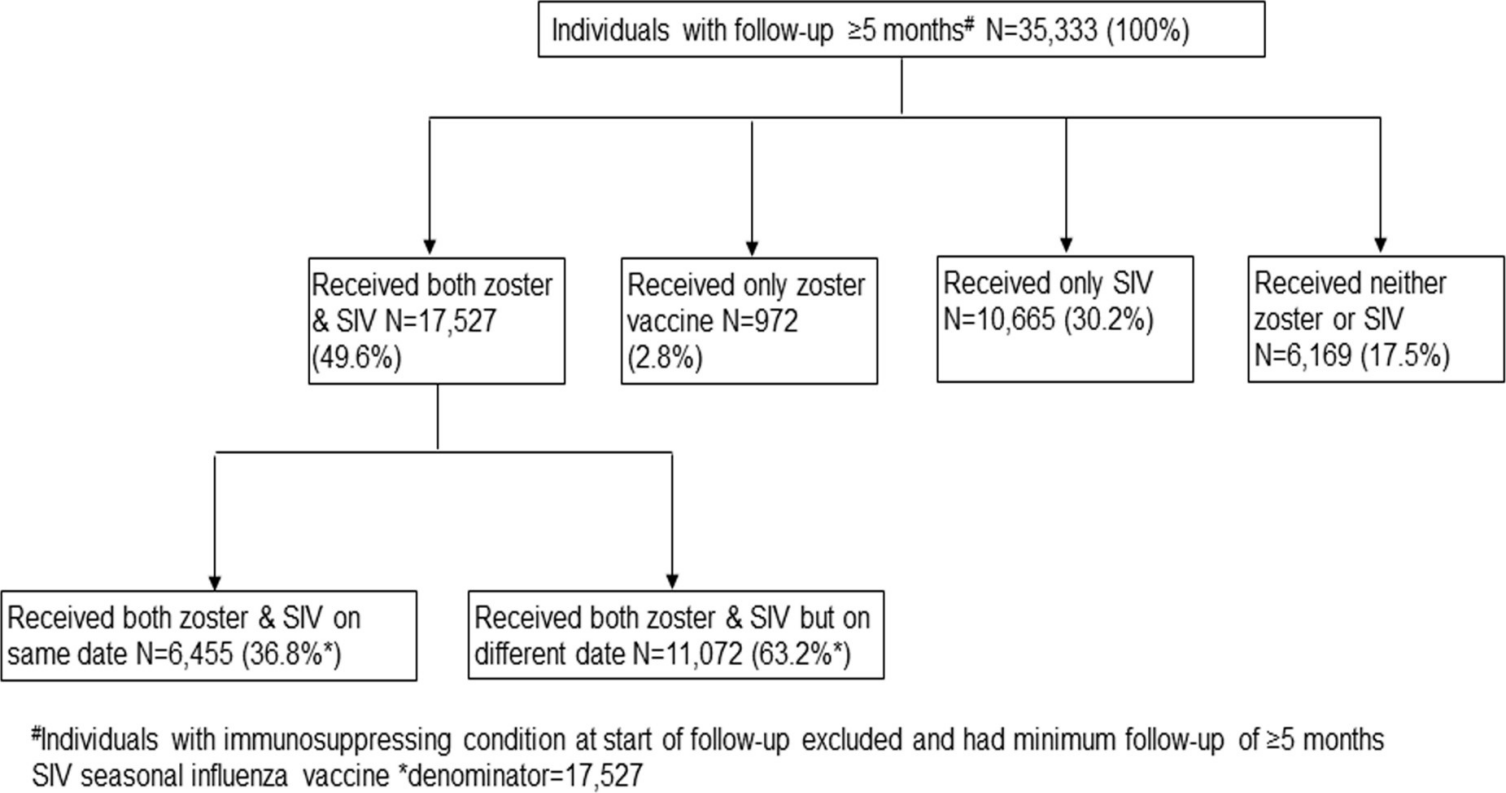
Zoster and seasonal influenza vaccine uptake amongst study participants.

Of the total participants considered for the primary analysis, zoster vaccine was administered to 18,499 (52.4%) individuals. Uptake amongst the main target group (those born in 1943) was 53.4% compared to 50.8% amongst the catch-up cohort (individuals born in 1934). Nearly half (n = 17,527) of the participants received both zoster vaccine and SIV (Fig 2); of these, only 36.8% (n = 6455) got both vaccines on same date (Fig 2), however the majority (86.8%, n = 16,066) received zoster vaccination during the influenza campaign period (September-March)[26] of 2013/2014 and 2014/2015. Amongst 79.8% (n = 28,192) of the participants who received SIV, 73.3% (n = 20,685) received SIV in both the 2013/2014 and 2014/2015 campaign periods while 22.4% (N = 6,323) received SIV only in 2013/2014, and 4.2% (N = 1,184) only in 2014/2015 season (data not shown).

A decision was made to drop marital status from the multivariable analyses, due to a large proportion of individuals with missing data for this variable. Thus, in the subsequent complete case analysis, only patients with missing ethnicity data (N = 3884, 11%) were excluded. This resulted in a final study population of 31,449 (89%) individuals, amongst whom the zoster vaccine uptake was 53.5% (Fig 1). Comparison of individuals included (n = 31,449) and excluded (n = 3884) from the complete case analysis due to missing ethnicity data is available in S7 Table. Briefly, excluded individuals were more likely to be in the main target cohort, and to be from less deprived patient- and practice-level deprivation areas, and were less likely to be care home residents, to have evidence that they were an immigrant or to have past history of zoster. The excluded group was also less likely to be vaccinated for zoster.

Time-varying exposures at the start and end of follow-up remained largely unchanged for 98.8% individuals included in the complete case analysis (S8 Table).

#### a) Minimally adjusted model

In the analysis adjusted for *a priori* confounders (gender and year of birth), there was strong evidence of an association between higher zoster vaccine uptake and male gender, with uptake 10% higher compared to females (Table 2). There was also evidence of lower vaccine uptake amongst the catch-up cohort, immigrants, those of non-White ethnicity, care home residents, those living alone and those not cohabiting, with reduced odds of between 12% (being in the catch-up cohort) to 46% (Black ethnicity) (all p values <0.001, Table 2)). There was also strong evidence for a linear trend (p for trend <0.0001) for decreasing vaccine uptake with increasing patient-LSOA-level deprivation (IMD) score, the most deprived group having 34% lower odds of uptake compared to the least deprived group. Non-linear decreases in uptake were seen for practices in more deprived areas (Table 2).

**Table 2:** Multivariable analyses- social factors associated with zoster vaccine uptake complete case analysis: Individuals with minimum follow-up of 5 months^a^.

		Minimally adjusted for year of birth & gender OR (95% CI)	P value~ (PT)	Model 1 additionally adjusted for immigration status & ethnicity OR (95% CI)	P value~	Model 2 additionally adjusted for patient-LSOA-level IMD OR (95% CI)	P value~ (PT)	Model 3 adjusted for all variables unless indicated OR (95% CI)	P value~ (PT)
Gender	Male	1.10 (1.05–1.15)	0.0001	1.09 (1.05–1.14)	0.0001	1.09 (1.04–1.14)	0.0001	1.08 (1.04–1.13)	0.0005
	Female	1		1		1		1	
Year of birth	1943 (main target group)	1		1		1		1	
	1934 (catch-up cohort)	0.88 (0.84–0.92)	<0.0001	0.88 (0.84–0.92)	<0.0001	0.87 (0.84–0.91)	<0.0001	0.89 (0.85–0.93)	<0.0001
Immigration status	Not immigrant	1		1		1		1	
	Immigrant	0.71 (0.59–0.85)	0.0002	0.91 (0.75–1.11)	0.36	0.94 (0.77–1.15)	0.55	0.94 (0.77–1.14)	0.52
Ethnicity	White	1		1		1		1	
	South Asian	0.70 (0.60–0.82)	<0.0001	0.73 (0.61–0.86)	<0.0001	0.73 (0.62–0.86)	<0.0001	0.72 (0.61–0.85)	<0.0001
	Black	0.54 (0.44–0.67)		0.55 (0.44–0.67)		0.61 (0.49–0.75)		0.61 (0.49–0.75)	
	Other	0.58 (0.45–0.75)		0.59 (0.46–0.76)		0.60 (0.47–0.77)		0.61 (0.47–0.78)	
	Mixed	0.61 (0.40–0.94)		0.61 (0.40–0.94)		0.63 (0.41–0.96)		0.62 (0.40–0.96)	
Patient-LSOA-level IMD^#^	Least deprived	1		Not in model		1		1	
	2	0.92 (0.86–0.98)	<0.0001			0.92 (0.86–0.98)	<0.0001	0.92 (0.87–0.98)	<0.0001
	3	0.85 (0.79–0.90)	(<0.0001)			0.85 (0.80–0.91)	(<0.0001)	0.86 (0.81–0.92)	(<0.0001)
	4	0.77 (0.72–0.83)				0.78 (0.73–0.84)		0.80 (0.74–0.86)	
	Most deprived	0.66 (0.61–0.71)				0.67 (0.62–0.73)		0.69 (0.64–0.75)	
Practice-LSOA-level IMD	Least deprived	1		Not in model		Not in model#		Not in model#	
	2	0.76 (0.71–0.82)	<0.0001						
	3	0.86 (0.80–0.93)							
	4	0.94 (0.87–1.01)							
	Most deprived	0.81 (0.75–0.87)							
Care home*	No	1		Not in model		Not in model		1	
	Yes	0.66 (0.58–0.74)	<0.0001					0.64 (0.57–0.73)	<0.0001
Living alone*	Not living alone	1		Not in model		Not in model		1	
	Yes living alone	0.85 (0.81–0.89)	<0.0001					0.85 (0.81–0.90)	<0.0001
Cohabiting*	No	0.73 (0.70–0.77)	<0.0001	Not in model		Not in model		Not in model#	
	Yes	1							

^a^Those with immunosuppressing condition at start of follow-up excluded (Number of patients = 31449 vaccine uptake = 16,838 (53.5%)) OR odds ratio CI confidence interval ~ likelihood ratio test PT P value for linear trend LSOA Lower-layer Super Output Area IMD Index of Multiple Deprivation ^**#**^ 19 patients with missing patient-LSOA-level IMD were replaced with practice-LSOA-level IMD

*at start of follow-up

# excluded due to multicollinearity issues

#### b) Multivariable analyses

After additionally adjusting the minimally adjusted model for immigration status and ethnicity (Multivariable Model 1, Table 2), no appreciable changes in effect estimates were observed, except lower uptake amongst immigrants was no longer apparent after adjustment for ethnicity, with no evidence of collinearity between the two variables. As patient- and practice-LSOA-level IMD were considered to be correlated, and as social factors relevant at an individual level were more of interest, only patient-LSOA-level IMD was added to Model 2. No noticeable changes in effect estimates between Model 1 and Model 2 were observed, and the strong evidence of linear trend of lower uptake with increasing deprivation score seen in minimally adjusted analysis was still evident (p<0.001) in this model. Similarly, living alone status and cohabitation were closely correlated, and so living alone was added to the final multivariable model (Model 3), along with care home residence. Again, the previously observed associations of lower uptake with living alone and residing in care home persisted in this model; individuals living alone and those residing in care home had 15% and 36% decreased odds of uptake, respectively (Table 2). The effect estimates for other variables were unchanged.

There was evidence that the effect of male gender, patient-LSOA-level IMD and care home residence varied with age (Table 3). Analyses showed that the increase in vaccine uptake among males was restricted to the catch-up cohort, and that the effects of care home status and (to a lesser extent) increasing deprivation on lower vaccine uptake were more marked in the catch-up cohort compared to the routine cohort.

**Table 3:** Interaction between age and social factors.

	Gender		Total N (column %)	Zoster vaccinations N (row %)	Stratum-specific adjusted^#^ OR for zoster vaccination (95%CI)	P-value for interaction*
Main target group	Males		9059 (48.4%)	4977 (54.9%)	1.00 (0.95–1.06)	<0.0001
	Females		9677 (51.6%)	5303 (54.8%)	1	
Catch-up cohort	Males		5786 (45.5%)	3156 (54.5%)	1.22 (1.13–1.31)	
	Females		6927 (54.5%)	3402 (49.1%)	1	
	Immigration status		Total N (column %)	Zoster vaccinations N (row %)	Stratum-specific adjusted^#^ OR for zoster vaccination (95%CI)	P-value for interaction*
Main target group	Not immigrant		18432 (98.4%)	10139 (55%)	1	0.93
	Immigrant		304 (1.6%)	141 (46.4%)	0.93 (0.73–1.19)	
Catch-up cohort	Not immigrant		12527 (98.5%)	6478 (51.7%)	1	
	Immigrant		186 (1.5%)	80 (43%)	0.95 (0.70–1.29)	
	Ethnicity		Total N (column %)	Zoster vaccinations N (row %)	Stratum-specific adjusted^#^ OR for zoster vaccination (95%CI)	P-value for interaction*
Main target group	White		17878 (95.4%)	9907 (55.4%)	1	0.73
	South Asian		423 (2.3%)	202 (47.8%)	0.75 (0.61–0.91)	
	Black		211 (1.1%)	79 (37.4%)	0.55 (0.41–0.72)	
	Other		170 (0.9%)	69 (40.6%)	0.57 (0.42–0.78)	
	Mixed		54 (0.3%)	23 (42.6%)	0.61 (0.35–1.05)	
Catch-up cohort	White		12174 (95.8%)	6337 (52.1%)	1	
	South Asian		246 (1.9%)	102 (41.5%)	0.68 (0.52–0.89)	
	Black		169 (1.3%)	68 (40.2%)	0.70 (0.51–0.96)	
	Other		92 (0.7%)	38 (41.3%)	0.67 (0.44–1.03)	
	Mixed		32 (0.3%)	13 (40.6%)	0.64 (0.32–1.31)	
	Patient-LSOA-level IMD		Total N (column %)	Zoster vaccinations N (row %)	Stratum-specific adjusted^#^ OR for zoster vaccination (95%CI)	P-value for interaction*
Main target group	Least deprived		4781 (25.5%)	2766 (57.9%)	1	0.07
	2		4514 (24.1%)	2520 (55.8%)	0.93 (0.85–1.00)	
	3		4033 (21.5%)	2226 (55.2%)	0.91 (0.83–0.99)	
	4		3160 (16.9%)	1658 (52.5%)	0.83 (0.76–0.91)	
	Most deprived		2248 (12%)	1110 (49.4%)	0.75 (0.68–0.83)	
Catch-up cohort	Least deprived		3261 (25.7%)	1837 (56.3%)	1	
	2		3208 (25.2%)	1732 (54%)	0.92 (0.83–1.01)	
	3		2677 (21.1%)	1342 (50.1%)	0.80 (0.72–0.88)	
	4		2119 (16.7%)	1024 (48.3%)	0.76 (0.68–0.84)	
	Most deprived		1448 (11.4%)	623 (43%)	0.62 (0.55–0.70)	
	Care home residence	Total N (column %)		Zoster vaccinations N (row %)	Stratum-specific adjusted^#^ OR for zoster vaccination (95%CI)	P-value for interaction*
Main target group	No	18217 (97.2%)		10021 (55%)	1	0.0008
	Yes	519 (2.8%)		259 (49.9%)	0.80 (0.67–0.95)	
Catch-up cohort	No	12097 (95.2%)		6328 (52.3%)	1	
	Yes	616 (4.8%)		230 (37.3%)	0.53 (0.45–0.63)	
	Living alone	Total N (column %)		Zoster vaccinations N (row %)	Stratum-specific adjusted^#^ OR for zoster vaccination (95%CI)	P-value for interaction*
Main target group	Not living alone	14005 (74.7%)		7860 (56.1%)	1	0.22
	Yes living alone	4731 (25.3%)		2420 (51.2%)	0.83 (0.78–0.89)	
Catch-up cohort	Not living alone	8791 (69.1%)		4618 (52.5%)	1	
	Yes living alone	3922 (30.9%)		1940 (49.5%)	0.89 (0.82–0.96)	

# Final Model 3 from Table 2 (Number of patients = 31449 vaccine uptake = 16,838 (53.5%)) OR odds ratio CI confidence interval

*likelihood ratio test LSOA Lower-layer Super Output Area IMD Index of Multiple Deprivation

### Sensitivity analyses

There were no appreciable differences between the baseline characteristics of individuals with follow-up for the entire 2-year study period and those included in primary analysis (follow-up period of ≥5 months) (Table 1). The results of multivariable analyses for those with longer follow up were similar to those from primary analysis except there was some attenuation in the association of ethnicity with vaccine uptake; individuals of Other ethnicities had 31% reduced odds (versus 39% in primary analysis) of uptake compared to those of White ethnicity in this model (Table 4).

**Table 4:** Sensitivity analyses: Social factors associated with zoster vaccine uptake complete case analysis (Model 3).

Variables		Primary analysis N = 31,449 vaccine uptake: n = 16,838		Sensitivity analysis^a^ N = 18,669 vaccine uptake: n = 11027		Sensitivity analysis^b^ N = 31,449 vaccine uptake: n = 16,838		Sensitivity analysis^c^ N = 31,449 vaccine uptake: n = 16,838		Sensitivity analysis^d^ N = 31,130 vaccine uptake: n = 16,707		Sensitivity analysis^e^ N = 31,449 vaccine uptake: n = 16,838		Sensitivity analysis^f^ N = 31,449 vaccine uptake: n = 16,838	
		OR (95% CI)	P value~ (PT)	OR (95% CI)	P value~ (PT)	OR (95% CI)	P value~ (PT)	OR (95% CI)	P value~ (PT)	OR (95% CI)	P value~ (PT)	OR (95% CI)	P value~ (PT)	OR (95% CI)	P value~ (PT)
Gender	Male	1.08 (1.04–1.13)	0.0005	1.10 (1.03–1.16)	0.003	1.07 (1.02–1.12)	0.003	1.08 (1.04–1.13)	0.0005	1.09 (1.04–1.14)	0.0003	1.08 (1.04–1.13)	0.0005	1.09 (1.04–1.14)	0.0003
	Female	1		1		1		1		1		1		1	
Year of birth	1943	1		1		1		1		1		1		1	
	1934	0.89 (0.85–0.93)	<0.0001	0.92 (0.86–0.98)	0.006	0.89 (0.85–0.93)	<0.0001	0.89 (0.85–0.93)	<0.0001	0.89 (0.85–0.93)	<0.0001	0.89 (0.85–0.94)	<0.0001	0.89 (0.85–0.93)	<0.0001
Immigrant	No	1		1		1		1		1		1		1	
	Yes	0.94 (0.77–1.14)	0.52	0.82 (0.62–1.08)	0.16	0.94 (0.77–1.15)	0.56	0.92 (0.75–1.12)	0.4	0.96 (0.79–1.18)	0.72	0.93 (0.77–1.14)	0.51	0.94 (0.77–1.15)	0.54
Ethnicity	White	1		1		1		1		1		1		1	
	South Asian	0.72 (0.61–0.85)	<0.0001	0.73 (0.57–0.94)	0.03	0.76 (0.64–0.89)	<0.0001	0.71 (0.60–0.84)	<0.0001	0.73 (0.62–0.86)	<0.0001	0.72 (0.61–0.85)	<0.0001	0.72 (0.61–0.86)	<0.0001
	Black	0.61 (0.49–0.75)		0.85 (0.62–1.15)		0.64 (0.52–0.79)		0.55 (0.45–0.68)		0.59 (0.48–0.74)		0.61 (0.49–0.75)		0.62 (0.50–0.76)	
	Other	0.61 (0.47–0.78)		0.69 (0.49–0.97)		0.62 (0.48–0.79)		0.60 (0.47–0.78)		0.62 (0.48–0.80)		0.60 (0.47–0.78)		0.61 (0.47–0.78)	
	Mixed	0.62 (0.40–0.96)		0.84 (0.47–1.50)		0.63 (0.41–0.98)		0.61 (0.40–0.93)		0.58 (0.38–0.91)		0.62 (0.40–0.96)		0.63 (0.41–0.96)	
Patient- LSOA-level IMD	Least deprived	1		1		1		Not in model#		1		1		1	
	2	0.92 (0.87–0.98)	<0.0001	0.84 (0.77–0.92)	<0.0001	0.93 (0.87–0.99)	<0.0001			0.94 (0.88–1.00)	<0.0001	0.92 (0.87–0.98)	<0.0001	0.92 (0.87–0.98)	<0.0001
	3	0.86 (0.81–0.92)	(<0.0001)	0.81 (0.74–0.88)	(<0.0001)	0.87 (0.81–0.92)	(<0.0001)			0.86 (0.81–0.92)	(<0.0001)	0.86 (0.81–0.92)	(<0.0001)	0.86 (0.81–0.92)	(<0.0001)
	4	0.80 (0.74–0.86)		0.74 (0.67–0.81)		0.81 (0.76–0.87)				0.80 (0.75–0.86)		0.80 (0.75–0.86)		0.80 (0.74–0.86)	
	Most deprived	0.69 (0.64–0.75)		0.67 (0.60–0.75)		0.72 (0.66–0.78)				0.69 (0.64–0.75)		0.70 (0.65–0.76)		0.69 (0.64–0.75)	Contd.
Practice- LSOA-level IMD	Least deprived	Not in model#		Not in model#		Not in model#		1		Not in model#		Not in model#		Not in model#	
	2							0.77 (0.72–0.83)	<0.0001						
	3							0.87 (0.81–0.94)							
	4							0.97 (0.90–1.04)							
	Most deprived							0.83 (0.77–0.90)							
Care home*	No	1		1		1		1		1		1		1	
	Yes	0.64 (0.57–0.73)	<0.0001	0.68 (0.58–0.80)	<0.0001	0.69 (0.61–0.78)	<0.0001	0.63 (0.56–0.71)	<0.0001	0.64 (0.57–0.73)	<0.0001	0.63 (0.57–0.70)	<0.0001	0.64 (0.57–0.72)	<0.0001
Living alone*	Not living alone	1		1		Not in model#		1		1		1		1	
	Yes living alone	0.85 (0.81–0.90)	<0.0001	0.84 (0.79–0.90)	<0.0001			0.83 (0.79–0.88)	<0.0001	0.86 (0.82–0.91)	<0.0001	0.85 (0.81–0.89)	<0.0001	0.86 (0.81–0.90)	<0.0001
Cohabiting*	No	Not in model#		Not in model#		1		Not in model#		Not in model#		Not in model#		Not in model#	
	Yes					1.30 (1.25–1.36)	<0.0001								
History of zoster*	No	Not in model		Not in model		Not in model		Not in model		Not in model		Not in model		1	
	Yes													1.12 (1.04–1.20)	0.002

OR odds ratio CI confidence interval PT P value for trend ~ likelihood ratio test

^a^ restricted to individuals with follow-up of 24 months

^b^ Including cohabitation instead of living alone in multivariable analysis LSOA Lower-layer Super Output Area

^c^ Including practice-LSOA- level IMD instead of patient-LSOA-level IMD

^d^ Excluding patients with immunosuppressing condition at end of follow-up (n = 1835) instead of at start of follow-up and excluding those with follow-up <5months

^e^ Care home status at end of follow-up

^f^ includes history of zoster in the model

*determined at start of follow-up IMD Index of Multiple Deprivation

# excluded due to multicollinearity issues

Substitution of cohabitation status instead of living alone, practice- LSOA-level IMD instead of patient-LSOA-level IMD, excluding individuals with immunosuppressive conditions at the end as opposed to start of follow-up (S9 Table) and determining care home status at the end instead of start of follow-up (Table 4 sensitivity analyses) did not change the findings. Individuals with a past history of zoster had 12% higher odds of uptake (Table 4), but inclusion of past zoster in the multivariable model made little difference to the other effect estimates.

To assess the impact of excluding individuals from the complete case analysis, a further exploratory analysis was conducted, by re-running the minimally adjusted analysis for the entire study population (N = 35,333) with follow-up ≥5 months including those with missing data on ethnicity (S10 Table). Comparing the minimally adjusted model with that of primary analysis revealed no noticeable differences in effect estimates. As the zoster vaccination programme is delivered through primary care in England, the effect of clustering at practice-level was also examined in an additional sensitivity analysis, using a multivariable (Model 3 in Table 2) logistic regression model with random effects (S11 Table). Although there was evidence for clustering within practices (with a p value <0.001 for the likelihood ratio test of an intra-cluster correlation coefficient of 0), the effect estimates in the random effects model were very similar to those from the primary analysis except for weaker evidence of reduced uptake among two of the non-White ethnicities (South Asian and Mixed ethnicity, S11 Table).

### Inadvertent zoster vaccinations

Of the 19,330 in the total study population who received vaccination (Fig 1), 3% (n = 596) received zoster vaccine whilst immunosupressed.[4] Of these 596 patients, 28 (4.7%) patients had more than one immunosuppressive condition at the time of vaccination. The maximum number of immunosuppressive conditions at time of vaccination was three. The most common immunosuppressive condition (n = 445) during which the patients received zoster vaccine was cancer chemotherapy or radiotherapy, followed by patients taking other immunosuppressive medications (n = 69), patients with leukemia, lymphoma, myeloma, other plasma cell dyscrasias (n = 49), treatment with immunosuppressive dose of oral corticosteroid (n = 28), cellular immune deficiency (n = 25), solid organ transplant (n = 9) and HIV (n = 1). None of the patients received zoster vaccination during the immunosuppressive phase of a stem cell or bone marrow transplant.

## Discussion

To the best of our knowledge, this is the first study to quantify the inequalities in the uptake of zoster vaccine, administered in a national vaccination programme, using anonymised electronic health records. This large 2-year population based study from England revealed that lower zoster vaccine uptake was independently associated with being a part of the catch-up cohort, non-White ethnicity, residing in a care home, living alone, and not cohabiting (living as a couple). A graded inverse association of patient-level deprivation with vaccine uptake was also observed. Lower uptake was also seen amongst females in the catch-up cohort, and the effects of care home residence and deprivation were more marked among the older catch-up group.

### Strengths and limitations

Strengths of the study include the large sample size, and linkages to hospitalisation data which provided additional information about socio-demographic factors as well as zoster vaccine contraindications. For multivariable analysis, a hierarchical approach using conceptual framework, enabled appropriate interpretation of effect estimates.[28] The NHS zoster vaccination programme is administered via general practice only and thus capture of vaccination is likely to be good. Additionally, our previous methodological study investigating ascertainment of socio-demographic factors in linked CPRD data showed that the distribution of factors such as ethnicity, not living alone, cohabitation and care home residence for individuals aged ≥65 years was comparable to the distribution in the 2011 English Census.[15]

The study limitations include potential misclassification of both socio-demographic factors and the zoster vaccination recording. Our categorisation of individuals without a relevant codes for a binary socio-demographic variable as not having the characteristic could have introduced errors; if this misclassification was non-differential with respect to vaccine uptake, this may have underestimated the effect estimates in this study. However, our previous methodological study showed good capture of socio-demographic factors in CPRD data when compared to the 2011 English Census.[15] Similarly, certain immunosuppressive treatments such as biological agents, which are mainly prescribed in a hospital setting, may not be completely captured in these primary care data. An additional issue is that some individuals may have changed exposure status over time. Ideally, person-time at risk to estimate vaccination rates would have been preferable but unavailability of complete dates of birth in CPRD precluded this ascertainment. However, factors ascertained at start of follow-up remained unchanged for 98.8%-99.9% individuals at the end of follow-up and sensitivity analyses to assess the impact of the time-varying factors reassuringly revealed similar results to the primary analysis. There was also potential for bias resulting from the complete case analysis (owing to missing ethnicity information) which led to exclusion of data from 11% of the study population. Further assessment of this revealed that effect estimates from the minimally adjusted models for individuals for the entire study population (after dropping ethnicity) had no appreciable differences to those obtained from the complete case analysis. Lack of recording of marital status prevented investigation of its association with zoster vaccine uptake, but other closely related variables for living arrangements such as cohabitation and living alone were available.

Other factors indicative of socio-economic status of an individual and associated with vaccine uptake amongst older individuals, as reported by our systematic review,[12] such as income, education, and occupation could not examined in this study as these factors were unavailable in CPRD. However, the association of deprivation using IMD quintiles for both patient- and practice-LSOA-level could be assessed in this study.

It is also feasible that individuals who see their GPs more frequently may have greater opportunity to be offered vaccination. Thus, it is possible that healthcare utilisation may be a potential mediating variable for the association between socio-demographic factors such as deprivation, male gender (in the catch-up cohort) or ethnicity and zoster vaccine uptake. If this were the case, adjusting for this mediating variable could attenuate the association between some of the socio-demographic factors and vaccine uptake.

Although we were able to examine whether there was evidence of clustering at practice-level, lack of information on practice-level variables (other than practice-LSOA-level-IMD) prevented further investigation of this. It is possible that variations in certain general practice-level characteristics, for example staffing levels, opening hours, and call/recall processes, could have affected implementation of the zoster vaccine programme for all zoster vaccine-eligible patients in that practice. These general practice-level data were unavailable in CPRD and therefore we could not determine to what extent the lower uptake we found in specific groups related to lack of opportunity to be vaccinated rather than individual choice not to come forward for vaccination. However, it is notable that practice-level clustering had little impact on most of the effect estimates studied other than some of the estimates for ethnicity. Future studies assessing the effects of these factors on vaccine uptake may provide a better understanding for the reasons of lower uptake amongst specific population groups.

### Comparison with other studies

Our finding of higher uptake in the main target group compared to the catch-up cohort reflects the findings from the national annual zoster vaccine coverage data for England over the same time period, and vaccine coverage among the individuals in our study who were followed up for the entire study duration (and thus had fuller capture of uptake of vaccination) is comparable to the coverage estimates in the national data.[6, 7] Higher uptake amongst males in the catch-up cohort observed in this study also was shown in the national data for 2013–2015 for reasons that are currently unexplained.[6, 7] This is in contrast to findings from North America that have reported a higher zoster vaccine uptake amongst females.[29–31] The majority of zoster vaccinations (87%) in this study occurred during the influenza season, suggesting that opportunistic targeting of the eligible population for zoster vaccine during SIV programme might have played a role. The national annual zoster vaccination data also supports this finding.[6, 7] Our finding of a linear relationship between increasing level of deprivation and lower zoster vaccine uptake also confirms the earlier analyses of the national data, which found a similar trend but was restricted to examining deprivation at the general practice-level;[14] studies from the US and Canada have also reported the lower zoster vaccine amongst individuals with lower income.[30–32] Higher zoster vaccine uptake amongst individuals of White ethnicity, as seen here, has been reported from other zoster vaccine studies from high income countries.[14, 30, 32–35]

Several of our findings are novel with respect to zoster vaccine uptake. These include lower uptake among individuals who were living alone or not cohabiting; this echoes studies showing lower uptake of seasonal influenza vaccine among those living alone in older European populations.[12] Our finding that immigration status was not independently associated with zoster vaccine uptake after adjusting for ethnicity is in contrast with previous findings of lower uptake among immigrant populations for seasonal influenza and pneumococcal vaccine in Spain and Israel, although none of these studies adjusted additionally for ethnicity.[36–43] The lower uptake of zoster vaccine among care home residents adds to a number of studies investigating uptake of other vaccines such as influenza and pneumococcal vaccines which have reported higher and lower uptake respectively, amongst care home residents.[44–48]

### Interpretation of findings and implications

This study demonstrates that in a public funded healthcare system, vaccination inequalities exist during a crucial period of programme initiation, and identifies socio-demographic groups that could be targeted with tailored interventions to increase zoster vaccine uptake. Of particular interest is the finding of lower uptake among care home residents; we have shown recently that individuals in care homes are at higher risk of developing zoster,[49] and so are a group with possible double health inequity (of both zoster burden and zoster vaccine uptake). Lower vaccine uptake among these residents could be due to lack of awareness amongst care home staff about the newly introduced programme and issues around getting consent. The reasons cited for differential (higher or lower) uptake of other vaccines among care home residents have included presence of vaccination polices in care homes, staff awareness, vaccination consent from the residents, location and care home ownership (public versus private).[44, 47, 48] The potential double health inequity amongst care home residents highlights a need for more rigorous targeting of these individuals to mitigate health inequality.

Similarly, targeting of older individuals who live alone may be needed to encourage zoster vaccination. Individuals cohabiting or living with their relatives may be more motivated by their social networks to get vaccinated. Secondly, higher disease awareness amongst these individuals, by witnessing the debilitating effect of zoster in their relatives, may also increase uptake. This was examined in a US study that reported higher zoster vaccine uptake amongst individuals in the three months after occurrence of zoster in their partners, reflecting a short term effect of disease awareness.[50] However, our finding that adjustment for a past personal history of zoster made little difference to effect estimates of social factors suggests that social networks may have a longer-lasting effect on encouraging vaccine uptake in older individuals, or that the occurrence of zoster in partners versus self may have a different effect on uptake. The lower uptake of vaccine among those of non-White ethnicity, but the attenuation of this association after restricting to individuals with longer follow up, suggests that there might be delay in uptake amongst some ethnic groups. There may be a lack of zoster disease awareness among some ethnic groups because of lower lifetime risk of zoster, which may be due to genetic causes, social mixing patterns that limit contacts with varicella and thus boosting of varicella-zoster virus immunity, and late onset of varicella among those born overseas.[33, 50, 51] Alternatively, the lower uptake may reflect existing healthcare inequalities. The lack of association of immigration status with zoster vaccine uptake after adjusting for ethnicity could be due to confounding by ethnicity, or simply to lack of power to detect an effect—the number of individuals identified as immigrants in the study population was relatively small (1.4%; n = 512) of which zoster vaccine uptake was observed amongst only 229 individuals. It is also feasible that in England, where national zoster vaccination programme is available free-of-charge, vaccination inequalities were not observed for older immigrant populations.

## Conclusions

This population-based cohort study provides evidence of inequalities in zoster vaccine uptake in the period immediately after the introduction of a national vaccination programme, identifying a wide range of socio-demographic determinants of uptake of zoster vaccine. This work should encourage further research into the reasons why specific socio-demographic groups are less likely to receive zoster vaccine, and effective planning and implementation of specific interventions to target these socio-demographic groups to mitigate vaccination inequalities amongst older individuals. Factors that are currently poorly recorded in routinely collected data, such as religion, education and income, should inform policy drivers such as the sustainability and transformation partnerships to incentivise better recording of these factors and/or facilitate other data linkages for comprehensive knowledge and future interventions to improve overall health and wellbeing of older populations. As care home residents are both less likely to receive zoster vaccine and are at higher risk of zoster, improving the uptake of zoster vaccination in this group will also mitigate inequalities in zoster burden.

## Supporting information

S1 Table Code list: Zoster vaccine. (DOCX)

S2 Table Code list social factors and immunosuppressive conditions. (DOCX)

S3 Table Code list seasonal influenza vaccine. (DOCX)

S4 Table Dose and duration criteria for immunosuppressive conditions/therapies. (DOCX)

S5 Table Zoster and post-herpetic neuralgia codes. (DOCX)

S6 Table Hierarchical conceptual framework and interpretation of effect estimates. (DOCX)

S7 Table Baseline characteristics of patients excluded from primary complete case analysis due to missing ethnicity and those included in analysis with complete covariate data. (DOCX)

S8 Table Changes in time varying factors at start and end follow-up. (DOCX)

S9 Table Changes in immunosuppressive therapy/ condition during follow-up. (DOCX)

S10 Table Multivariable analysis: Social factors associated with zoster vaccine uptake: Primary complete case analysis excluding ethnicity. (DOCX)

S11 Table Social factors associated with zoster vaccine uptake: Accounting for clustering at general practice level. (DOCX)

S12 Table The RECORD statement–checklist of items, extended from the STROBE statement that should be reported in observational studies using routinely collected health data. (DOCX)

S1 Text Details of determining zoster vaccination status. (DOCX)

S1 Fig Decision flow chart for ascertaining zoster vaccine status. (DOCX)
