# Supplementary material for: Zoster vaccination inequalities: A population based cohort study using linked data from the UK Clinical Practice Research Datalink
Source: PLoS One. 2018 Nov 15;13(11):e0207183. doi: 10.1371/journal.pone.0207183 (PMC6237346; doi:10.1371/journal.pone.0207183)
Supplement: S1 Table — (DOCX) [file pone.0207183.s001.docx]

**S1 Table** **Code list: zoster vaccine**

| medcode | readcode | readterm |
| --- | --- | --- |
| 106904 | 65FY.00 | Herpes zoster vaccination |
| 106593 | 65FY.11 | Shingles vaccination |
| 107067 | 65FY000 | Herpes zoster vaccination given by other health care provide |
| 106948 | 68Nv.00 | No consent for herpes zoster vaccination |
| 106946 | 8I2r.00 | Herpes zoster vaccination contraindicated |
| 106947 | 8IEl.00 | Herpes zoster vaccination declined |
| 107061 | 9Nig.00 | Did not attend herpes zoster vaccination |
| 108895 | U60K600 | [X]Herpes zoster vacc caus adverse effects therapeutic use |

| prodcode | Productname |
| --- | --- |
| 47327 | Zostavax vaccine powder and solvent for suspension for injection 0.65ml pre-filled syringes (sanofi pasteur MSD Ltd) |
| 48314 | Shingles (Herpes Zoster) vaccine (live) powder and solvent for suspension for injection 0.65ml pre-filled syringes |

| immtype | Description |
| --- | --- |
| 88 | Shingles |
| 91 | Shingles OHP |
