# Supplementary material for: Zoster vaccination inequalities: A population based cohort study using linked data from the UK Clinical Practice Research Datalink
Source: PLoS One. 2018 Nov 15;13(11):e0207183. doi: 10.1371/journal.pone.0207183 (PMC6237346; doi:10.1371/journal.pone.0207183)
Supplement: S3 Table — (DOCX) [file pone.0207183.s003.docx]

**S3 Table** **Code list seasonal influenza vaccine**

| **medcode** | **readcode** | **readterm** |
| --- | --- | --- |
| 6 | 65E..00 | Influenza vaccination |
| 10821 | 68NV.00 | Influenza vacc consent given |
| 12336 | ZV04800 | [V]Influenza vaccination |
| 13025 | U60K400 | [X]Influenza vaccine causing adverse effects therapeutic use |
| 21123 | ZV04811 | [V]Flu - influenza vaccination |
| 49070 | F034G00 | Post influenza vaccination encephalitis |
| 94301 | 65E0.00 | First pandemic influenza vaccination |
| 95092 | 65E1.00 | Second pandemic influenza vaccination |
| 97941 | 65E2.00 | Influenza vaccination given by other healthcare provider |
| 98047 | 68Nr.00 | Consent given for pandemic influenza vaccination |
| 98183 | 65E9.00 | PANDEMRIX - first influenza A (H1N1v) 2009 vaccination given |
| 98184 | 65EA.00 | PANDEMRIX - second influenza A (H1N1v) 2009 vaccination give |
| 98203 | 65EB.00 | PANDEMRIX - 1st flu A (H1N1v) 2009 vac by othr hlth provider |
| 98217 | 65E3.00 | 1st pandemic influenza vacc give by other healthcare providr |
| 98234 | 65E5.00 | CELVAPAN - first influenza A (H1N1v) 2009 vaccination given |
| 98302 | 65E6.00 | CELVAPAN - second influenza A (H1N1v) 2009 vaccination given |
| 98303 | 65E8.00 | CELVAPAN - 2nd flu A (H1N1v) 2009 vacc by othr hlth provider |
| 98304 | 65EC.00 | PANDEMRIX - 2nd flu A (H1N1v) 2009 vac by othr hlth provider |
| 98306 | 65E4.00 | 2nd pandemic influenza vacc give by other healthcare providr |
| 98449 | 65E7.00 | CELVAPAN - 1st flu A (H1N1v) 2009 vacc by othr hlth provider |
| 99801 | 68Nt.00 | Consent given for influenza A subtype H1N1 vaccination |
| 104688 | 65ED.00 | Seasonal influenza vaccination |
| 104958 | 68NV000 | Consent given for seasonal influenza vaccination |
| 105077 | 65E2000 | Seasonal influenza vaccin given by other healthcare provider |
| 105195 | 65ED000 | Seasonal influenza vaccination given by pharmacist |
| 106994 | 65EE000 | Administration of first intranasal influenza vaccination |
| 106995 | 65EE100 | Administration of second intranasal influenza vaccination |
| 107156 | 65EE.00 | Administration of intranasal influenza vaccination |
| 107297 | 65ED100 | Administration of first intranasal seasonal influenza vacc |
| 107315 | 65E0000 | Administration of first intranasal pandemic influenza vacc |
| 107352 | 65ED300 | Administration of second intranasal seasonal influenza vacc |
| 107413 | 65E2100 | First intranasal seasonal flu vacc gvn by othr hlthcare prov |
| 107573 | 65ED200 | Seasonal influenza vaccination given while hospital inpt |
| 107646 | 65E1000 | Administration of second intranasal pandemic influenza vacc |
| 107730 | 65E2200 | Secnd intranasal seasonal flu vacc gvn by othr hlthcare prov |
| 108772 | 65E3000 | First intranasal pndmc influenza vcc gvn othr hlthcare prvdr |
| 110182 | 65E2400 | 1st intramuscular seasonal influenza vacc given by other HCP |
| 110219 | 65E2300 | 2nd intramuscular seasonal influenza vacc given by other HCP |
| 110854 | 65ED400 | Administration of first inactivated seasonal influenza vacc |

| **prodcode** | **productname** |
| --- | --- |
| 398 | Influenza inactivated split virion Vaccination (Aventis Pasteur MSD) |
| 639 | Influenza vaccine (split virion, inactivated) suspension for injection 0.5ml pre-filled syringes |
| 834 | Begrivac vaccine suspension for injection 0.5ml pre-filled syringes (Novartis Vaccines and Diagnostics Ltd) |
| 922 | Influenza inactivated surface antigen Vaccination |
| 1329 | Fluvirin vaccine suspension for injection 0.5ml pre-filled syringes (Novartis Vaccines and Diagnostics Ltd) |
| 2139 | Fluarix vaccine suspension for injection 0.5ml pre-filled syringes (GlaxoSmithKline UK Ltd) |
| 2552 | Influvac Sub-unit vaccine suspension for injection 0.5ml pre-filled syringes (Abbott Healthcare Products Ltd) |
| 2601 | Mfv-ject Vaccination (Aventis Pasteur MSD) |
| 7951 | FLUVIRIN AQUEOUS ML VAC |
| 9710 | Agrippal vaccine suspension for injection 0.5ml pre-filled syringes (Novartis Vaccines and Diagnostics Ltd) |
| 10030 | Inflexal V vaccine suspension for injection 0.5ml pre-filled syringes (Janssen-Cilag Ltd) |
| 11824 | Enzira vaccine suspension for injection 0.5ml pre-filled syringes (Pfizer Ltd) |
| 13595 | Fluzone Vaccination (Aventis Pasteur MSD) |
| 16585 | Viroflu vaccine suspension for injection 0.5ml pre-filled syringes (Janssen-Cilag Ltd) |
| 18612 | Mastaflu vaccine suspension for injection 0.5ml pre-filled syringes (Masta Ltd) |
| 23251 | FLUVIRIN PRE-FILLED SYRINGE |
| 24779 | Influenza inactivated split virion Paediatric vaccination |
| 27407 | Imuvac vaccine suspension for injection 0.5ml pre-filled syringes (Abbott Healthcare Products Ltd) |
| 30156 | Invivac vaccine suspension for injection 0.5ml pre-filled syringes (Abbott Healthcare Products Ltd) |
| 30198 | Influenza inactivated split virion Vaccination (sanofi pasteur MSD Ltd) |
| 32391 | Influenza vaccine (surface antigen, inactivated) suspension for injection 0.5ml pre-filled syringes (Novartis Vaccines and Diagnostics Ltd) |
| 38421 | Influenza inactivated split virion Vaccination (Evans Vaccines Ltd) |
| 40760 | Influenza vaccine (split virion, inactivated) 15microgram strain suspension for injection 0.1ml pre-filled syringes |
| 40876 | Influenza vaccine (split virion, inactivated) 9microgram strain suspension for injection 0.1ml pre-filled syringes |
| 41150 | Pandemrix vaccine emulsion and suspension for emulsion for injection (GlaxoSmithKline UK Ltd) |
| 41168 | Influenza H1N1 vaccine (split virion, inactivated, adjuvanted) emulsion and suspension for emulsion for injection |
| 41240 | Influenza H1N1 vaccine (whole virion, Vero cell derived, inactivated) suspension for injection |
| 41925 | Celvapan (H1N1) vaccine (whole virion, Vero cell derived, inactivated) suspension for injection (Baxter Healthcare Ltd) |
| 43825 | Intanza 15microgram strain vaccine suspension for injection 0.1ml pre-filled syringes (sanofi pasteur MSD Ltd) |
| 43827 | Intanza 9microgram strain vaccine suspension for injection 0.1ml pre-filled syringes (sanofi pasteur MSD Ltd) |
| 44759 | INFLUENZA PRE-FILLED SYRINGE |
| 45661 | Influenza vaccine (split virion, inactivated) suspension for injection 0.5ml pre-filled syringes (Pfizer Ltd) |
| 47932 | Fluenz vaccine nasal suspension 0.2ml unit dose (AstraZeneca UK Ltd) |
| 48085 | Influenza inactivated split virion Vaccination (Chiron UK Ltd) |
| 48658 | Influenza vaccine (split virion, inactivated) suspension for injection 0.5ml pre-filled syringes (sanofi pasteur MSD Ltd) |
| 48740 | Influenza vaccine (surface antigen, inactivated) suspension for injection 0.5ml pre-filled syringes |
| 49716 | Influenza vaccine (surface antigen, inactivated, virosome) suspension for injection 0.5ml pre-filled syringes |
| 51087 | Optaflu vaccine suspension for injection 0.5ml pre-filled syringes (Novartis Vaccines and Diagnostics Ltd) |
| 51289 | Influenza vaccine (live attenuated) nasal suspension 0.2ml unit dose |
| 54677 | Preflucel vaccine suspension for injection 0.5ml pre-filled syringes (Baxter Healthcare Ltd) |
| 57140 | Influenza vaccine (live attenuated) nasal suspension 0.2ml unit dose |
| 57401 | Influvac Desu vaccine suspension for injection 0.5ml pre-filled syringes (Abbott Healthcare Products Ltd) |
| 57678 | Fluenz vaccine nasal suspension 0.2ml unit dose (AstraZeneca UK Ltd) |
| 57917 | Fluarix Tetra vaccine suspension for injection 0.5ml pre-filled syringes (GlaxoSmithKline UK Ltd) |
| 61580 | Influenza vaccine (split virion, inactivated) suspension for injection 0.25ml pre-filled syringes |
| 61792 | Fluenz Tetra vaccine nasal suspension 0.2ml unit dose (AstraZeneca UK Ltd) |
| 61898 | Influenza vaccine (split virion, inactivated) suspension for injection 0.5ml pre-filled syringes (A A H Pharmaceuticals Ltd) |
| 63690 | Inflexal V suspension for injection 0.5ml pre-filled syringes (sanofi pasteur MSD Ltd) |
| 65205 | FluMist Quadrivalent vaccine nasal suspension 0.2ml unit dose (AstraZeneca UK Ltd) |

| Code | Immunisation type |
| --- | --- |
| 4 | FLU |
| 71 | PFLUGEN |
| 72 | PFLUGSK |
| 73 | PFLUGSKO |
| 74 | PFLUGS |
| 75 | PFLUBAXO |
| 76 | PFLUBAX |
| 78 | PFLUGENO |
| 84 | FLUSOHP |
| 85 | FLUSPHARMA |
| 89 | FLUSIN |
| 97 | FLUSINOHP |
| 100 | FLUSIMOHP |
