# Supplementary material for: Zoster vaccination inequalities: A population based cohort study using linked data from the UK Clinical Practice Research Datalink
Source: PLoS One. 2018 Nov 15;13(11):e0207183. doi: 10.1371/journal.pone.0207183 (PMC6237346; doi:10.1371/journal.pone.0207183)
Supplement: S4 Table — (DOCX) [file pone.0207183.s004.docx]

**S4 Table** **Dose and duration criteria for immunosuppressive conditions/therapies**

The immunosuppressive conditions included: leukaemia, lymphoma, myeloma, other plasma cell dyscrasias, stem cell transplant, bone marrow transplant, solid organ transplants, Human Immunodeficiency Virus infection and cellular immune deficiency. The immunosuppressive treatments identified were: biological therapies, azathioprine, methotrexate, 6-mercaptopurine, steroids, other immunosuppressive agents such as tacrolimus, other disease modifying anti-rheumatic drugs such as ciclosporin, cancer chemotherapy and radiotherapy. The code lists used for identifying these conditions in both CPRD & HES data are presented in S2 Table. The immunosuppressive dose criteria and the duration of period of immunosuppression for these conditions/treatments (required for time-updating these variables to identify immunosuppressive periods and thus immune status at the time of vaccine receipt) are described in table below.

| **Immunosuppressive condition or therapy** | **Dose criteria if applicable** | **Period of immune-suppression prior to first medical record** | **Period of immune-suppression after every record** |
| --- | --- | --- | --- |
| Lymphoma, myeloma, other plasma cell dyscrasias, leukemia, bone marrow transplant stem cell transplant | Not applicable | - | 24 months |
| Cellular immune deficiency, solid organ transplants and HIV | Not applicable | - | For life |
| Azathiaprine | Immune-suppressed at dose of ≥50mg/daily assuming the worst  case scenario of TPMT carrier[1, 2] | 3 months^#^ | 3 months |
| Methotrexate | Immune-suppressed at dose of>25mg per week (>3.57 mg/day) | 3 months^#^ | 3 months |
| 6-mercaptopurine | Immune-suppressed at dose of ≥45mg/daily (assuming the worst case scenario of TPMT carrier) [1, 2] | 3 months^#^ | 3 months |
| Other immune-suppressive agents | Immune-suppressed any dose | 3 months^#^ | 3 months |
| Biological agents (e.g. Anti-TNF therapy) | Immune-suppressed any doses | 3 months^#^ | 12 months |
| Other DMARDs e.g. mycophenolate, leflunomide | Immune-suppressed any dose | 3 months^#^ | 3 months |
| Injectable or oral steroids | Immune-suppressed : >40 mg/daily for >7 days OR >20mg/ daily for >14 days [3] | 3 months^#^ | 3 months |
| Cancer chemotherapy or radiotherapy | Immune-suppressed any dose | 3 months^#^ | 12 months |

^#^ as the therapy is generally initiated in hospitals TNF tumour necrosis factor TPMT thiopurine methyltransferase DMARD disease modifying anti-rheumatic drugs

1. British Society of Gastroenterology. Azathioprine / 6 mercaptopurine [16/02/2017]. Available from: <www.bsg.org.uk/pdf_word_docs/aza_ibd_dr.doc>.

2. Ford LT, Berg JD. Thiopurine S-methyltransferase (TPMT) assessment prior to starting thiopurine drug treatment; a pharmacogenomic test whose time has come. J Clin Pathol. 2010;63(4):288-95.

3. Public Health England. Chapter 28a : Shingles . In: Immunisation against infectious disease. The Green Book. 2016 [16/04/2017]. Available from: <https://www.gov.uk/government/publications/shingles-herpes-zoster-the-green-book-chapter-28a>.
