# Supplementary material for: Zoster vaccination inequalities: A population based cohort study using linked data from the UK Clinical Practice Research Datalink
Source: PLoS One. 2018 Nov 15;13(11):e0207183. doi: 10.1371/journal.pone.0207183 (PMC6237346; doi:10.1371/journal.pone.0207183)
Supplement: S6 Table — (DOCX) [file pone.0207183.s006.docx]

**S6 Table** **Hierarchical conceptual framework and interpretation of effect estimates**

(based on [1])

| **Hierarchical models** | **Explanatory variables** | **Interpretation of effect estimates** |
| --- | --- | --- |
| `Minimally’ adjusted model | Each explanatory variable adjusted in-turn for *a priori* confounders: year of birth and gender | Effect estimate of each variable adjusted for *a* *priori* confounders. |
| Model-1*^^^ | Ethnicity +immigration status^^^ with *a priori* confounders | Effects of ethnicity and immigration status adjusted for each other and *a priori* confounders |
| Model-2* | Model-1+ patient-LSOA-level deprivation^#^ | 1. (i) Effects of ethnicity and immigration status not mediated via deprivation and adjusted for each other and *a priori* confounders   (ii) Effect of patient-LSOA-level deprivation adjusted for *a priori* confounders, ethnicity and immigration status |
| Model-3* | Model-2 + rest of the explanatory variables~ | (i) Effect of ethnicity and immigration status not mediated via deprivation and other explanatory variables~ *  (ii) Effect of deprivation not mediated via other explanatory variables~*  (iii) Effect of other explanatory variables~ * |

*all variables in the model adjusted for each other and *a priori* confounders: year of birth, sex and calendar period ^^^ethnicity and immigration status examined for multicollinearity LSOA Lower-layer Super Output Area ^#^ patient-LSOA-level and practice-LSOA-level deprivation were considered to be correlated therefore only patient-LSOA-level deprivation used ~ care home residence, living alone status and cohabitation status (living alone and cohabitation examined for multicollinearity)

1. Victora CG, Huttly SR, Fuchs SC, Olinto MT. The role of conceptual frameworks in epidemiological analysis: a hierarchical approach. Int J Epidemiol. 1997;26(1):224-7. PubMed PMID: 9126524.
