# Supplementary material for: Zoster vaccination inequalities: A population based cohort study using linked data from the UK Clinical Practice Research Datalink
Source: PLoS One. 2018 Nov 15;13(11):e0207183. doi: 10.1371/journal.pone.0207183 (PMC6237346; doi:10.1371/journal.pone.0207183)
Supplement: S7 Table — (DOCX) [file pone.0207183.s007.docx]

**S7 Table** **Baseline characteristics of patients excluded from primary complete case analysis due to missing ethnicity and those included in analysis with complete covariate data**

| **Variables** |  | **Excluded from complete case analysis^1^ due to missing data on ethnicity**  **N=3884, vaccine uptake=1661 (42.8%)**  Age (years) at current registration date: median (IQR) 48.9 (36.4-58.2), mean (range) 46.3 (0-79) | | | **Included in complete case analysis^1^**  **N=31449, vaccine uptake=16,838 (53.5%)**  Age (years) at current registration date: median (IQR) 52.9 (40.4-62.8), mean (range) 50 (0-79) | | |
| --- | --- | --- | --- | --- | --- | --- | --- |
|  |  |  |  |  |  |  |  |
|  |  |  |  |  |  |  |  |
|  |  | Total (column %) | Not received zoster vaccine (row %) | Received zoster vaccine (row %) | Total (column %) | Not received zoster vaccine (row %) | Received zoster vaccine (row %) |
| **Gender** | Male | 1788 (46%) | 1062 (59.4%) | 726 (40.6%) | 14845 (47.2%) | 6712 (45.2%) | 8133 (54.8%) |
|  | Female | 2096 (54%) | 1161 (55.4%) | 935 (44.6%) | 16604 (52.8%) | 7899 (47.6%) | 8705 (52.4%) |
| **Year of birth** | 1943 (main target group) | 2722 (70.1%) | 1550 (56.9%) | 1172 (43.1%) | 18736 (59.6%) | 8456 (45.1%) | 10280 (54.9%) |
|  | 1934 (catch-up cohort) | 1162 (29.9%) | 673 (57.9%) | 489 (42.1%) | 12713 (40.4%) | 6155 (48.4%) | 6558 (51.6%) |
| **Immigration status** | Not immigrant | 3862 (99.4%) | 2209 (57.2%) | 1653 (42.8%) | 30959 (98.4%) | 14342 (46.3%) | 16617 (53.7%) |
|  | Immigrant | 22 (0.6%) | 14 (63.6%) | 8 (36.4%) | 490 (1.6%) | 269 (54.9%) | 221 (45.1%) |
| **Patient-LSOA-level IMD** | Least deprived | 1271 (32.7%) | 644 (50.7%) | 627 (49.3%) | 8042 (25.6%) | 3439 (42.8%) | 4603 (57.2%) |
|  | 2 | 970 (25%) | 552 (56.9%) | 418 (43.1%) | 7722 (24.6%) | 3470 (44.9%) | 4252 (55.1%) |
|  | 3 | 810 (20.9%) | 494 (61%) | 316 (39%) | 6710 (21.3%) | 3142 (46.8%) | 3568 (53.2%) |
|  | 4 | 549 (14.1%) | 341 (62.1%) | 208 (37.9%) | 5279 (16.8%) | 2597 (49.2%) | 2682 (50.8%) |
|  | Most deprived | 284 (7.3%) | 192 (67.6%) | 92 (32.4%) | 3696 (11.8%) | 1963 (53.1%) | 1733 (46.9%) |
| **Practice-LSOA- level IMD** | Least deprived | 976 (25.1%) | 476 (48.8%) | 500 (51.2%) | 5208 (16.6%) | 2229 (42.8%) | 2979 (57.2%) |
|  | 2 | 868 (22.3%) | 514 (59.2%) | 354 (40.8%) | 7111 (22.6%) | 3513 (49.4%) | 3598 (50.6%) |
|  | 3 | 844 (21.7%) | 510 (60.4%) | 334 (39.6%) | 6563 (20.9%) | 3048 (46.4%) | 3515 (53.6%) |
|  | 4 | 559 (14.4%) | 350 (62.6%) | 209 (37.4%) | 5896 (18.7%) | 2617 (44.4%) | 3279 (55.6%) |
|  | Most deprived | 637 (16.4%) | 373 (58.6%) | 264 (41.4%) | 6671 (21.2%) | 3204 (48%) | 3467 (52%) |
| **Care home*** | No | 3819 (98.3%) | 2192 (57.4%) | 1627 (42.6%) | 30314 (96.4%) | 13965 (46.1%) | 16349 (53.9%) |
|  | Yes | 65 (1.7%) | 31 (47.7%) | 34 (52.3%) | 1135 (3.6%) | 646 (56.9%) | 489 (43.1%) |
| **Living alone*** | Not living alone | 2729 (70.3%) | 1469 (53.8%) | 1260 (46.2%) | 22796 (72.5%) | 10318 (45.3%) | 12478 (54.7%) |
|  | Yes living alone | 1155 (29.7%) | 754 (65.3%) | 401 (34.7%) | 8653 (27.5%) | 4293 (49.6%) | 4360 (50.4%) |
| **Cohabiting*** | No | 1754 (45.2%) | 1103 (62.9%) | 651 (37.1%) | 13598 (43.2%) | 6933 (51%) | 6665 (49%) |
|  | Yes | 2130 (54.8%) | 1120 (52.6%) | 1010 (47.4%) | 17851 (56.8%) | 7678 (43%) | 10173 (57%) |
| **History of zoster*** | No | 3524 (90.7%) | 2037 (57.8%) | 1487 (42.2%) | 27795 (88.4%) | 12996 (46.8%) | 14799 (53.2%) |
|  | Yes | 360 (9.3%) | 186 (51.7%) | 174 (48.3%) | 3654 (11.6%) | 1615 (44.2%) | 2039 (55.8%) |

^1^ Those with immune-suppressing condition at start of follow-up excluded with minimum follow-up >=5 months IQR interquartile range LSOA Lower-layer Super Output Area IMD index of multiple deprivation * at start of follow-up
