# Supplementary material for: Zoster vaccination inequalities: A population based cohort study using linked data from the UK Clinical Practice Research Datalink
Source: PLoS One. 2018 Nov 15;13(11):e0207183. doi: 10.1371/journal.pone.0207183 (PMC6237346; doi:10.1371/journal.pone.0207183)
Supplement: S8 Table — (DOCX) [file pone.0207183.s008.docx]

**S8 Table** **Changes in time varying factors at start and end follow-up**

| Variables | Patients with information available N (%) | Patients with same information at start and end of follow-up N (%) | Patients with different exposure information at start and end of follow-up N (%) |
| --- | --- | --- | --- |
| Living alone | 31449 (100%) | 31427 (99.9%) | 22 (0.1%) |
| Cohabiting | 31449 (100%) | 31439 (99.97%) | 10 (0.03%) |
| Care home | 31449 (100%) | 31080 (98.8%) | 369 (1.2%) |
| Marital status | 19930 (63.3%) | 19895 (99.8%) | 35 (0.2%) |
