# Supplementary material for: Zoster vaccination inequalities: A population based cohort study using linked data from the UK Clinical Practice Research Datalink
Source: PLoS One. 2018 Nov 15;13(11):e0207183. doi: 10.1371/journal.pone.0207183 (PMC6237346; doi:10.1371/journal.pone.0207183)
Supplement: S9 Table — (DOCX) [file pone.0207183.s009.docx]

**S9 Table Changes in immunosuppressive therapy/ condition during follow-up**

Out of the total study population N=39120, 2818 individuals had record(s) of immunosuppressive condition/therapy during follow-up. An individual might have >1 condition at a given time; similarly one individual may be immunosuppressed at different time points during the follow-up. Number of individuals with immunosuppressive condition/ therapy record at start of follow-up was N=1494, at end of follow-up was N=1835. Individuals with a code(s) during the middle of the follow-up and not at the start or end of the follow-up was 377.

| **Immunosuppressive therapy or condition** | **Total number* of patients with one or more immunosuppressive condition(s) or taking immunosuppressive therapy (therapies) at any time during follow-up: N=2818 (Total study population=39120)** | | | | |
| --- | --- | --- | --- | --- | --- |
|  | **Total number of individuals with the condition*** | **Patients with condition at start of follow-up & not at end-follow-up (row%)** | **Patients with condition at end of follow-up & not at start of follow-up (row%)** | **Patients with condition at both start & end of follow-up (row%)** | **Patients with condition only during middle of follow-up not at start or end of follow-up (row%)** |
| Oral corticosteroids | 194 | 46 (23.7%) | 45 (23.2%) | 26 (13.4%) | 77 (39.7%) |
| Other immune- suppressive drugs~ | 317 | 48 (15.1%) | 51 (16.1%) | 195 (61.5%) | 23 (7.3%) |
| Lymphoma, myeloma, other plasma cell dyscrasias & leukemia | 399 | 75 (18.8%) | 166 (41.6%) | 158 (39.6%) | 0 (0%) |
| Stem cell transplant, bone marrow transplant | 12 | 5 (41.7%) | 5 (41.7%) | 2 (16.7%) | 0 (0%) |
| Cancer radio/chemotherapy | 2082 | 525 (25.2%) | 788 (37.8%) | 446 (21.4%) | 323 (15.5%) |
| Solid organ transplant | 60 | 0 (0%) | 6 (10%) | 54 (90%) | 0 (0%) |
| Cellular immune deficiency | 132 | 0 (0%) | 57 (43.2%) | 75 (56.8%) | 0 (0%) |
| HIV | 15 | 0 (0%) | 0 (0%) | 15 (100%) | 0 (0%) |

*an individual may have >1 condition/therapy at a given time ~includes azathioprine, biological therapy, methotrexate, 6-mercaptopurine, other immunosuppressant such as tacrolimus, sirolimus, and other disease-modifying antirheumatic drugs: ciclosporin, mycophenolate, leflunomide
