# Supplementary material for: Zoster vaccination inequalities: A population based cohort study using linked data from the UK Clinical Practice Research Datalink
Source: PLoS One. 2018 Nov 15;13(11):e0207183. doi: 10.1371/journal.pone.0207183 (PMC6237346; doi:10.1371/journal.pone.0207183)
Supplement: S10 Table — (DOCX) [file pone.0207183.s010.docx]

**S10 Table Social factors associated with zoster vaccine uptake: primary complete case analysis excluding ethnicity**

Number of patients=35,333 vaccine uptake=18,499

| **Variables** |  | **Minimally adjusted for year of birth & gender OR (95% CI)** | **P value~**  **(PT)** |
| --- | --- | --- | --- |
| **Gender** | Male | 1.07 (1.02-1.11) | 0.002 |
|  | Female | 1 |  |
| **Year of birth** | 1943 (main target group) | 1 |  |
|  | 1934 (catch-up cohort) | 0.90 (0.87-0.94) | <0.0001 |
| **Immigration status** | Not immigrant | 1 |  |
|  | Immigrant | 0.73 (0.62-0.88) | 0.0005 |
| **Patient-LSOA-level IMD^$^** | Least deprived | 1 |  |
|  | 2 | 0.91 (0.86-0.96) | <0.0001 (<0.0001) |
|  | 3 | 0.83 (0.78-0.89) |  |
|  | 4 | 0.77 (0.72-0.82) |  |
|  | Most deprived | 0.66 (0.61-0.71) |  |
| **Practice-LSOA-level IMD** | Least deprived | 1 |  |
|  | 2 | 0.76 (0.71-0.82) | <0.0001 |
|  | 3 | 0.84 (0.79-0.90) |  |
|  | 4 | 0.92 (0.85-0.98) |  |
|  | Most deprived | 0.81 (0.76-0.87) |  |
| **Care home*** | No | 1 |  |
|  | Yes | 0.71 (0.63-0.79) | <0.0001 |
| **Living alone*** | Not living alone | 1 |  |
|  | Yes living alone | 0.82 (0.78-0.86) | <0.0001 |
| **Cohabiting*** | No | 0.72 (0.69-0.75) | <0.0001 |
|  | Yes | 1 |  |

OR odds ratio CI confidence interval PT P value for trend ~ likelihood ratio test LSOA Lower-layer Super Output Area IMD index of multiple deprivation **^$^**26 patients with missing patient-LSOA-level IMD were replaced with practice-LSOA-level IMD *at start of follow-up
