# Supplementary material for: Zoster vaccination inequalities: A population based cohort study using linked data from the UK Clinical Practice Research Datalink
Source: PLoS One. 2018 Nov 15;13(11):e0207183. doi: 10.1371/journal.pone.0207183 (PMC6237346; doi:10.1371/journal.pone.0207183)
Supplement: S11 Table — (DOCX) [file pone.0207183.s011.docx]

**S11 Table Social factors associated with zoster vaccine uptake: accounting for clustering at general practice level**

| Variables |  | Primary analysis (Model 3)  N=31,449 vaccine uptake: n=16,838 | | Sensitivity analysis (Model 3) after taking into account clustering at practice level^#^  N=31,449; vaccine uptake: n=16,838 | |
| --- | --- | --- | --- | --- | --- |
|  |  | OR (95% CI) | P value~ | OR (95% CI) | P value~ |
| Gender | Male | 1.08 (1.04-1.13) | 0.0005 | 1.08 (1.03-1.14) | 0.0009 |
|  | Female | 1 |  | 1 |  |
| Year of birth | 1943 | 1 |  | 1 |  |
|  | 1934 | 0.89 (0.85-0.93) | <0.0001 | 0.87 (0.82-0.91) | <0.0001 |
| Immigrant | No | 1 |  | 1 |  |
|  | Yes | 0.94 (0.77-1.14) | 0.52 | 0.88 (0.70-1.10) | 0.27 |
| Ethnicity | White | 1 |  | 1 |  |
|  | South Asian | 0.72 (0.61-0.85) | <0.0001 | 0.85 (0.71-1.03) | 0.0001 |
|  | Black | 0.61 (0.49-0.75) |  | 0.66 (0.52-0.84) |  |
|  | Other | 0.61 (0.47-0.78) |  | 0.67 (0.51-0.88) |  |
|  | Mixed | 0.62 (0.40-0.96) |  | 0.65 (0.41-1.04) |  |
| Patient-LSOA-level IMD | Least deprived | 1 |  | 1 |  |
|  | 2 | 0.92 (0.87-0.98) | <0.0001 | 0.92 (0.85-0.99) | <0.0001 |
|  | 3 | 0.86 (0.81-0.92) |  | 0.82 (0.76-0.89) |  |
|  | 4 | 0.80 (0.74-0.86) |  | 0.76 (0.70-0.83) |  |
|  | Most deprived | 0.69 (0.64-0.75) |  | 0.71 (0.64-0.78) |  |
| Care home* | No | 1 |  | 1 |  |
|  | Yes | 0.64 (0.57-0.73) | <0.0001 | 0.61 (0.54-0.70) | <0.0001 |
| Living alone* | Not living alone | 1 |  | 1 |  |
|  | Yes living alone | 0.85 (0.81-0.90) | <0.0001 | 0.84 (0.80-0.89) | <0.0001 |

# primary analysis taking into account clustering at practice level (logistic regression model with random effects to adjust for clustering at practice level (N=277 in England) OR odds ratio CI confidence interval ~ likelihood ratio LSOA Lower-layer Super Output Area IMD Index of Multiple Deprivation
