# Supplementary material for: Zoster vaccination inequalities: A population based cohort study using linked data from the UK Clinical Practice Research Datalink
Source: PLoS One. 2018 Nov 15;13(11):e0207183. doi: 10.1371/journal.pone.0207183 (PMC6237346; doi:10.1371/journal.pone.0207183)
Supplement: S1 Text — (DOCX) [file pone.0207183.s013.docx]

**S1 Text Details of determining zoster vaccination status**

The Immunisation file includes an additional ‘vaccination status’ field which provides information on whether the vaccine was advised, refused or given. Similarly, Read codes for declining, not consenting or not attending for zoster vaccination were identified (Appendix 1). To assign vaccination status to each individual, an algorithm was developed using the following hierarchy for different CPRD files (Appendix 3). The Therapy files were given the highest rank, as presence of a product code in therapeutic file indicated that a prescription was actually issued by the GP.[1] The second priority was assigned to the records in the Immunisation files with additional information about whether the vaccine was advised, refused or given. The next in the hierarchy was information obtained using the Read codes from the Clinical, Referral or the Test files. First individuals with conflicting vaccination information (i.e.simultaneous presence of receipt of zoster vaccination and refusal/declining/not attending/not consenting codes on same date) were excluded from the study (Appendix 3). For the remaining participants, individuals were considered to be vaccinated if they had the relevant product code in the Therapy file. The Immunisation files of individuals without vaccination information from the Therapy file were then examined for the presence of relevant immunisation type code. These individuals were considered to be vaccinated if they had relevant code and the status field of the Immunisation file was `given’. Finally, individuals without zoster vaccination information from the Therapy or Immunisation files and had relevant Read codes in their Clinical, Referral or Test files were considered to be vaccinated.

1. Clinical Practice Research Datalink. Clinical Practice Research Datalink [11/09/2017]. Available from: <https://www.cprd.com/intro.asp>.
