# Supplementary material for: Zoster vaccination inequalities: A population based cohort study using linked data from the UK Clinical Practice Research Datalink
Source: PLoS One. 2018 Nov 15;13(11):e0207183. doi: 10.1371/journal.pone.0207183 (PMC6237346; doi:10.1371/journal.pone.0207183)
Supplement: S1 Fig — (DOCX) [file pone.0207183.s014.docx]

**S1 Fig Decision flow chart for ascertaining zoster vaccine status**

Patient excluded from the study

Patients without conflicting codes on the same date

Presence of zoster specific product code for zoster vaccine in the Therapy file

Vaccinated

Patients with no zoster vaccination information from the Therapy file: If they had necessary immunisation type code for zoster vaccine and the status field was `given’

Vaccinated

Patients with no zoster vaccination information from the Immunisation file: If they had necessary Read codes for zoster vaccine in the Clinical, Referral or Test file

Vaccinated

Patients with conflicting codes on same date i.e. if zoster vaccination codes and codes for refusal/declining /did not attend/ no consent co-exited
